# Retinopathy and microcirculation in adult severe malaria

**DOI:** 10.1186/1475-2875-9-S2-I7

**Published:** 2010-10-20

**Authors:** Richard James Maude, Abdullah Abu Sayeed, Nicholas AV Beare, Prakaykaew Charunwatthana, Abul M Faiz, Amir Hossain, Emran Bin Yunus, Gofranul M Hoque, Mahtab Uddin Hasan, Nicholas J White, Nicholas PJ Day, Arjen M Dondorp

**Affiliations:** 1 Mahidol-Oxford Tropical Medicine Research Unit, Faculty of Tropical Medicine, Mahidol University, Bangkok 10400, Thailand; 2 Centre for Tropical Medicine, Nuffield Department of Clinical Medicine, CCVTM, University of Oxford, Churchil Hospital, Old Road, Oxford, OX3 7LJ, UK; 3 Division of Clinical & Surgical Sciences, University of Edinburgh, Royal Infirmary of Edinburgh, Edinburgh, EH16 4SA, UK; 4 Chittagong Medical College Hospital, Chittagong, Bangladesh; 5 St Paul's Eye Unit, Royal Liverpool University Hospital, Liverpool, UK; 6 Sir Salimullah Medical College, Dhaka, Bangladesh

## Introduction

A specific retinopathy has been described in African children with cerebral malaria, but in adults this has not been extensively studied. It has great potential as a diagnostic and prognostic tool and pathogenetic marker. Since the structure and function of the retinal vasculature greatly resembles the cerebral vasculature, study of retinal changes can reveal insights into the pathogenesis of cerebral malaria. Obstruction of microcirculatory blood flow is thought to be important in causing both malarial retinopathy and cerebral malaria.

## Methods

A detailed observational study of malarial retinopathy in Bangladeshi adults with P. falciparum malaria was performed using a portable retinal camera. The aims were to establish the prevalence, spectrum and time to resolution of retinal findings in malaria, their effect on visual function and to investigate the role of obstruction of microcirculatory blood flow in its pathogenesis. A second study aimed to establish if assessment of malarial retinopathy in adult malaria using ophthalmoscopy by non-ophthalmologists has clinical and prognostic significance.

For the first study, all Plasmodium falciparum smear positive adult patients admitted to Chittagong Medical College Hospital, Chittagong, Bangladesh, during the malaria seasons of 2008-2010 were eligible. Control groups were febrile encephalopathy, sepsis and healthy volunteers and children with malaria. Patients were photographed daily until discharge then weekly until complete resolution of retinal changes. In adults, severity of retinal findings was correlated with markers of microcirculatory blood flow obstruction (blood lactate and rectal capillary blood flow (Microscan)) and rheological factors important in microcirculatory obstruction (P. falciparum histidine rich protein 2 (PfHRP2) and red blood cell deformability (LORCA)).The second study recruited unselected patients with severe and uncomplicated falciparum malaria, vivax malaria and healthy controls. Retinal examinations were by two non-ophthalmologists, one using direct and the other indirect ophthalmoscopy.

## Results

Of 234 adults recruited in the first study, 51/60 (85%) with cerebral, 18/27 (67%) noncerebral and 28/59 (47%) uncomplicated malaria had retinal changes. Of the controls, 9/29 (31%) encephalopathy, 11/28 (39%) sepsis and 4/31 (13%) healthy volunteers had some degree of retinopathy, mostly mild. Moderate-severe retinopathy was found in 67% of those with fatal, 62% of cerebral, 41% noncerebral and 12% uncomplicated malaria, 14% encephalopathy, 0% sepsis and 0% healthy volunteers. Other than papilloedema, moderate-severe retinopathy was highly specific for malaria (98%), and for cerebral malaria in comatose patients (93%). Resolution of signs took median 14 days, and visual function 3-4, days. Severity of retinopathy correlated with severity of malaria, coma recovery time and markers of, and rheological factors important in, microcirculatory obstruction.

In 210 adults in the second study moderate-severe retinopathy as assessed by non-ophthalmologists using direct ophthalmoscopy was found to be an independent predictor of mortality in malaria. Although indirect ophthalmoscopy was more sensitive it provided minimal additional prognostic information compared to direct.

## Discussion

Malarial retinopathy is highly specific for malaria and was present in the majority of adults with severe disease, particularly cerebral and fatal, but was prominent in very few with uncomplicated disease. It thus has diagnostic and prognostic utility at the bedside, including when assessed by a non-specialist using direct ophthalmoscopy. Retinal findings were similar in adults and children. The strong correlation with markers of and rheological factors important in, microvascular obstruction suggest an important role of microvascular obstruction in the pathogenesis of both malarial retinopathy and cerebral malaria. Although malarial retinopathy lasted around two weeks, impairment of colour vision and acuity were only transient.

## Conclusions

Malarial retinopathy has potential as a bedside tool to aid diagnosis and prognosis in adults with malaria. Prominent retinal changes are highly suggestive of severe disease, particularly cerebral and fatal malaria. Findings in adults were very similar to those in children, both in the present study and in Africa. The correlation of severity of retinopathy with impaired microcirculatory blood flow suggest this process is central to the pathogenesis of both malarial retinopathy, and cerebral malaria. Studies using fluorescein angiography and magnetic resonance imaging to investigate this further are currently underway.

